# Frozen Section in the Management of Ovarian and Uterine Tumors: The Past 5 Years in a Tertiary Centre

**DOI:** 10.1055/s-0038-1668526

**Published:** 2018-08

**Authors:** Joana Aidos, Renata Veríssimo, Joana Almeida, Teresa Carvalho, Nuno Nogueira Martins, Francisco Nogueira Martins

**Affiliations:** 1 Department of Obstetrics and Gynecology, Centro Hospitalar Tondela Viseu, Viseu, Portugal; 2 Department of Pathological Anatomy, Centro Hospitalar Tondela Viseu, Viseu, Portugal

**Keywords:** intraoperative frozen section, ovarian tumors, uterine tumors, diagnóstico intraoperatório por congelação, tumores do ovário, tumores uterinos

## Abstract

**Objective** Intraoperative frozen section (IFS) is a valuable resource, and its use in gynecological pathology has not been sufficiently emphasized. The main goal of the present study is to evaluate the reliability and agreement rates between IFS and the final paraffin section (PS) and determine how reliable IFS is.

**Methods** A retrospective study of all IFSs performed on uterine tumors and suspicious adnexal masses between January 2012 and December 2016 (excluding metastases) at the department of obstetrics and gynecology of the Centro Hospitalar Tondela Viseu. Frozen versus permanent section diagnosis were compared regarding the histologic type of the tumor, and the depth of myometrial invasion.

**Results** A total of 286 cases were eligible for the present study, including 102 (35.7%) IFSs of uterine tumors, and 184 (64.3%) IFSs of ovarian tumors. The overall rate of deferred cases was 5.2% (15/286). The accuracy of the diagnosis in cases of endometrial carcinoma was 96.25% (77/80). Among the ovarian tumors, misdiagnoses occurred in 2 cases (1.1%), corresponding to a borderline tumor (serous type) and a clear cell intracystic adenocarcinoma.

**Conclusion** The IFS analysis plays an important role in selected situations and is associated to a high sensitivity and specificity in cases of ovarian and endometrial tumors. Its high accuracy is almost universally associated with the possibility of obtaining an optimal surgical treatment at the time of the first surgical approach.

## Abstract (pt)

**Resumo:**

**Objetivo** O diagnóstico intraoperatório por congelação é um recurso importante cujo uso em patologia ginecológica não tem sido suficientemente enfatizado. O objetivo do presente estudo foi avaliar as taxas de concordância entre o diagnóstico intraoperatório por congelação e o estudo anatomopatológico definitivo e determinar o quanto o diagnóstico intraoperatório por congelação é um método confiável.

**Métodos** Um estudo retrospectivo de todos os diagnósticos intraoperatórios por congelação realizados em tumores uterinos e massas anexiais suspeitas entre janeiro e 2012 e dezembro de 2016 (excluindo metástases) no serviço de ginecologia e obstetrícia do Centro Hospitalar Tondela Viseu. Comparação do diagnóstico intraoperatório por congelação com o resultado do estudo definitivo em relação ao tipo histológico do tumor e profundidade de invasão miometrial.

**Resultados** Um total de 286 casos foram elegíveis para o estudo, incluindo 102 (35.7%) tumores uterinos e 184 (64.3%) tumores ovarianos. A taxa global de casos deferidos foi de 5.2% (15/286). Entre os tumores uterinos, a acuidade de diagnóstico nos casos de carcinoma endometrial foi de 96.25% (77/80). Entre os tumores ovarianos, não se verificou concordância em 2 casos (1.1%), correspondendo a um tumor borderline do tipo seroso e a um adenocarcinoma de células claras intracístico.

**Conclusão** O diagnóstico intraoperatório por congelação apresenta-se com um importante papel em situações selecionadas, sendo acompanhado de elevada taxa de sensibilidade e especificidade para tumores endometriais e ovarianos. A sua elevada acuidade diagnóstica encontra-se associada à possibilidade de obter um tratamento cirúrgico adequado na primeira abordagem cirúrgica

## Introduction

Intraoperative frozen section (IFS) can prove to be a valuable resource, and its use in gynecological pathology has not been emphasized in the literature to the same degree as in other surgical fields. It is important for pathologists and surgeons to understand the role and limitations of IFS in gynecological oncology.^1^

Intraoperative frozen section plays a critical role in guiding gynecological tumor surgical procedures, determining whether the sample tissues are benign or malignant.^2^

The use of IFS is one of the most important steps in the operative management of suspicious adnexal masses. The IFS is usually requested to define the adequate surgical plan either by obtaining histological confirmation of suspected malignant or borderline primary ovarian tumors, or by ruling out malignancy in a suspicious adnexal mass.^3^

Regarding endometrial cancer (EC), IFS can potentially define the grade of the tumor, the depth of myometrial invasion, the histological type, and the existence or absence of cervical extension. Intraoperative frozen section is the only way to identify during surgery the subgroup of patients who are at a higher risk of extrauterine disease and therefore provide adequate guidance toward optimal surgical staging.^4^ ^5^

Relevant clinical information should include not only the previous history of malignancy, pathology reports, imaging studies such as ultrasonography or computed tomography (CT) scan and serum markers, but also the impression of the surgeon during the operation. Ovarian tumors represent the most common request site for intraoperative diagnosis, followed by endometrium, cervix, and vulva tumors.^2^

Intraoperative frozen section requires pathologists to possess excellent gross and microscopic diagnostic skills. The overdiagnosis can lead to unnecessary surgical intervention and increased morbidity and mortality. On the other hand, underdiagnosis is associated with tumor spread and need for additional surgeries.^3^

Surgeons should be aware that IFS diagnosis is based on the assessment of a few sections from the grossly most suspicious or representative portion of the tumor, while the final diagnosis is made after the evaluation of an internationally agreed standard of a minimum of one section per centimeter of maximal tumor diameter.^4^

An ideal IFS would have 100% accuracy for the evaluation of ovarian tumors. Nevertheless, the sensitivity and specificity for IFS in ovarian tumors range from 65 to 97% and from 97 to 100%, respectively.^6^ Large diameter, mucinous type and borderline tumors were shown to increase the discrepancy between IFS and the definitive pathological result.^6^ ^7^

Regarding endometrial tumors, the accuracy of IFS has been extensively discussed in the literature and concordance rates between IFS and paraffin section (PS) range from 68 to 95% for tumor grade and from 72 to 95% for depth of myometrial invasion.^4^ ^5^

Several investigators have suggested that IFS is an accurate and useful tool to guide intraoperative decision making for surgical staging in EC.^2^ ^6^ In contrast, several others have presented data that question the reliability of IFS.^7^ ^8^ Simple total hysterectomy (TH) plus bilateral salpingo-oophorectomy (BSO) remains a cornerstone for the management of EC, whereas the value of systematic lymphadenectomy is a matter of great debate.^9^ Many gynecologic oncologists have turned to IFS analysis as a means of determining which women should undergo lymph node sampling.^10^

At our institution, lymphadenectomy in the context of IFS is usually performed on patients with grade 1 or 2 endometrioid adenocarcinoma presenting with cervical involvement and/or more than 50% myometrial invasion in the IFS. Regarding ovarian tumors, the same is true for those with an IFS diagnosis of malignancy.

The aim of the present study is to evaluate the reliability and agreement rates between IFS and the final PS and to determine whether IFS is a reliable method for guiding the intraoperative decision-making or not, namely regarding the need for lymphadenectomy.

## Methods

In the present retrospective study, all IFSs performed on uterine and suspected ovarian tumors between January 2012 and December 2016 (excluding metastases) at the department of obstetrics and gynecology of the Centro Hospitalar Tondela Viseu were included. Frozen versus permanent section diagnoses were compared regarding the histologic type of the tumor, and the depth of myometrial invasion. During surgery, the uterus or/and adnexa were given to the pathologist for IFS examination. The pathologist determined the number of sections to be examined; assessed tumor size, grade, histologic subtype and, regarding endometrial tumors, the invasion of the cervix or of the external half of the myometrium. The results were reported to the operating surgeon while the patient was still anesthetized. The surgeon then determined the surgical conduct accordingly. In cases in which no definitive conclusions can be achieved by IFS, the pathology report is deferred. In most of these cases, a strong suspicion or possible exclusion of a particular diagnosis is given orally to the surgeon.

After reporting the results of the frozen section, the specimens were processed routinely for final PS analysis. Regarding ovarian tumors, the results of IFS and PS were divided in benign, borderline, and malignant. Regarding uterine tumors, the results were divided in benign (excluded) and malignant, including invasion of the cervix and of the external half of the myometrium. After that, the sensitivity, specificity, positive predictive value (PPV) and negative predictive value (NPV) were determined separately for benign, borderline, and malignant cases by considering the final PS diagnosis as the gold standard (Table 1).

**Table 1:** Characteristics of the patients

	Ovarian tumors (*n* = 184)	Uterine tumors (*n* = 102)
***Characteristic***		
Age mean	54.7	51
Age range	16–86	31–79
Body mass index mean (kg/m2)	26.2	32
Body mass index range	17–35	20–37
Postmenopausal	47.8%	90.2%

Age in years.

A total of 307 patients underwent hysterectomy and/or adnexectomy including IFS, 123 of which had a preoperative diagnosis of endometrioid adenocarcinoma of the uterus, complex atypical hyperplasia (CAH) or suspected cancer in hysteroscopy combined with inconclusive sampling.

Twenty-one patients were excluded from the study group: six cases of polypoid endometrium, three cases of adenomyosis, three cases of tubal benign tumors, two cases of endometrial hyperplasia without atypia, two cases of uterine fibroids, two cases of ovarian metastasis (one from the colon and another from the appendix), one case of cervical carcinoma, one case of parametrial fibroid and one case of ovarian varicocele.

Hence, 286 patients were eligible for analysis. These patients were analyzed with IFS to determine the need for complete surgical staging, including pelvic and para-aortic lymphadenectomy.

The approval of the institutional review board was obtained for the reviews of the medical records and pathologies of all patients. The ethical approval protocol was based on the World Medical Association's Declaration of Helsinki. No patient consent was required because the data were analyzed anonymously.

## Results

There were 102 (35.7%) uterine tumors and 184 (64.3%) ovarian tumors. The overall rate of deferred cases was 5.2% (15/286).

### Ovarian Tumors

A total of 184 patients were identified. The mean age was 54.7 ± 15.9 years old (range 16–86 years old). The majority of patients (84.7%) were > 40 years old and 47.8% (88) of them were postmenopausal.

The median serum concentration of the tumor marker CA125 was 20.6 (range 2.4–12548.0) IU/L (*n* = 133). The risk of ovarian malignancy algorithm (ROMA) was calculated in 100 cases with a high-risk result in 57 (57.0%) cases (*n* = 100).

The intraoperative frozen section of the 184 ovarian specimens revealed 75% (138) benign tumors, 1.6% (3) borderline tumors, 16.8% (31) malignant tumors, and 6.5% (12) deferred diagnoses. The final PS diagnoses revealed 77.1% (142) benign tumors, 4.9% (9) borderline tumors, and 17.9% (33) malignant tumors.

Of the 142 benign cases, 62 were non-neoplastic cystic lesions of the ovary, including endometriotic, follicular, and corpus luteal cysts. The most common benign neoplastic tumor was serous cystadenoma (28 cases), followed by mucinous cystadenoma (24 cases), mature cystic teratoma (21 cases), fibromas (6 cases) and Brenner tumor (1 case). Among the borderline neoplasms, 7 cases were of borderline serous and 2 cases of borderline mucinous neoplasms. The most common malignant tumors were serous carcinoma (17 cases) followed by endometrioid carcinoma (6 cases), clear cell carcinoma (5 cases), mucinous carcinoma (2 cases), malignant granulosa cells tumor (1 case), malignant epithelioid mesothelioma tumor (1 case) and Brenner malignant tumor (1 case).

Among the ovarian tumors, misdiagnosis occurred in 2 cases (1.1%), corresponding to a borderline tumor (serous type) and a clear cell intracystic adenocarcinoma, which were underdiagnosed as benign mucinous proliferation and borderline serous tumor, respectively, on the IFS.

In the 12 cases in which the IFS conclusions were deferred, the final diagnoses were: borderline mucinous tumor in 6 (5 serous and 1 mucinous type) cases, serous cystadenoma in 3 cases, mucinous cystadenoma in 1 case, teratoma in 1 case, and bilateral serous adenocarcinoma in 1 case (Table 2).

**Table 2:** Comparison between intraoperative frozen section and final histological diagnoses of ovarian masses

Frozen Diagnosis	Final histological diagnosis		
	Malignant	Borderline	Benign
**Malignant**	31	0	0
**Borderline**	1	2	0
**Benign**	0	1	137
Total	32	3	137

The sensitivity and specificity for benign, borderline, and malignant tumors were 100%, 66.7%, 96.9%, and 97.1%, 99.4%, 100%, respectively. The PPV and NPV for benign, borderline, malignant tumors were 99.3%, 66.7%, 100%, and 100%, 99.4% and 100%, respectively (Table 3).

**Table 3:** Sensitivity, specificity, positive predictive value and negative predictive value of the intraoperative frozen section for ovarian neoplasms

	Benign	Borderline	Malignant
**Sensitivity**	100%	66.7%	96.9%
**Specificity**	97.1%	99.4%	100%
**Positive predictive value**	99.3%	66.7%	100%
**Negative predictive value**	100%	99.4%	100%

Six patients (3.3%) had bilateral disease, and 21 patients (11.4%) had tumor spread beyond the ovaries.

All the patients with malignant intraoperative frozen section diagnosis underwent radical surgery, except four cases in which the tumor was unresectable.

### Uterine Tumors

A total of 102 patients were included. The mean age was 51.0 years old (range 31–79 years old). In the study group, 92 (90.2%) women were postmenopausal and 59 (64.1%) presented with postmenopausal bleeding. Ten patients were premenopausal (9.8%) and 6 of them (60%) had complaints of heavy and/or irregular menstrual bleeding.

As shown in Table 2, there were 64 patients with a preoperative diagnosis of endometrial carcinoma, 31 patients with CAH, 1 patient with complex hyperplasia without atypia, and 6 patients with suspected carcinoma at hysteroscopy with inconclusive biopsy. All of the patients were evaluated initially by IFS, and then by a definitive PS to determine the degree of concordance between IFS and PS.

A total of 26 patients underwent lymphadenectomy in the same operative process (18 bilateral pelvic and para-aortic, 6 bilateral pelvic and 2 unilateral pelvic). In five cases, there was metastatic disease in the pelvic lymph nodes, and in three cases both in the pelvic and para-aortic lymph nodes. One patient presented with metastatic disease in the periganglionar adipose tissue. In five cases, lymphadenectomy was not performed due to technical difficulties, such as lack of access, obesity or other significant comorbidities.

Concerning the tumor staging according to the International Federation of Gynecology and Obstetrics (FIGO, in the French acronym) classification system, 47 patients (58.7%) were included in stage IA, 19 patients (23.75%) in IB, 6 patients (7.5%) in IIA, 2 patients (2.5%) in IIIA and 6 patients (7.5%) in IIIC. From the study group, 75 (93.75%) women had type I and the remaining patients had type II endometrial carcinomas, including 2 with mixed adenocarcinoma (clear cell and serous); 1 with carcinosarcoma with cervical invasion; 1 with a mixed adenocarcinoma with neuroendocrine elements and 1 with a large cells neuroendocrine carcinoma (Table 4). The Accuracy of the Diagnosis of Endometrial Carcinoma was 96.25% (77/80).

**Table 4:** Correlation results between intraoperative frozen section and final histological diagnoses in endometrial tumors

	Number	Percentage
***Preoperative diagnosis***		
Cancer	64	62.7
Complex atypical hyperplasia	31	30.4
Complex hyperplasia without atypia	1	1.0
Suspected carcinoma	6	5.9
***IFS***		
Cancer	77	75.5
▪ Inner half	50	
▪ Outer half	27	
Without invasion	22	21.6
Deferred	3	2.9
	**Number**	**Percentage**
***Postoperative diagnosis (PS)***		
Cancer	80	78.4
▪ Inner half	50	
▪ Outer half	30	
Complex atypical hyperplasia	11	10.8
Benign	11	10.8

Abbreviations: IFS, intraoperative frozen section; PS, paraffin section.

The IFS correctly diagnosed the histologic type in 75 of 80 patients (93.75%). The depth of myometrial invasion was accurately diagnosed in 94.8% of the patients (73/77).

In the final PS, 75/80 tumors remained endometrioid adenocarcinomas, whereas 5 that were originally diagnosed as endometrioid adenocarcinoma in the IFS were changed to mixed histology in the PS. One was read as a grade 3 carcinosarcoma with cervical invasion, 1 as a grade 3 mixed papillary serous adenocarcinoma, 1 as a grade 3 mixed adenocarcinoma with neuroendocrine elements, 1 as large cells neuroendocrine tumor carcinoma, and 1 as a grade 3 mixed adenocarcinoma (clear cells and serous). Our correlation rate between IFS and PS for histological subtype was 93.75% (75/80). It is important to note that the intraoperative management was not affected in any of the five cases mentioned above (Table 5).

**Table 5:** Comparison between intraoperative frozen section and final histological diagnoses in terms of the presence or absence of endometrial cancer

IFS diagnosis	Final pathology result			Total
	No EC	EC Inner half	EC Outer half	
No EC	18	3	−	21
Deferred	3	−	−	3
EC				
▪ inner half myometrium	1	46	3	50
▪ outer half myometrium	−	1	27	28
Total	22	50	30	102

Abbreviations: EC, endometrial cancer; IFS, intraoperative frozen section.

### Complex Atypical Hyperplasia

A total of 31 patients had a preoperative diagnosis of CAH. In the IFS study, 11 cases were read as malignant, 17 as negative for malignant lesions and 3 with deferred diagnosis. In the postoperative PS, 11 cases were read as CAH, 7 cases were read as no residual disease, and 13 as endometrial cancer. It is important to note that 2 cases thought to be non-cancerous in the IFS were later determined to be cancerous in the final PS. These two cases had received a diagnosis of “no residual disease” in the IFS diagnosis. They were non-invasive grade 1 cancers, in which the discrepancy was not relevant to the surgical management, and they were staged according to the FIGO classification system as IA.

## Discussion

### Intraoperative Frozen Section in Ovarian Tumors

Ovarian cancer is the third more common malignant tumor, and its incidence has increased, especially in younger women.^11^

The clinical diagnosis of ovarian malignancy is challenging due the difficulty in obtaining a histological diagnosis before the definitive treatment.^12^

The optimal surgical management of ovarian tumors depends very much on their correct categorization as benign, borderline or malignant. The need for an additional surgical procedure may arise from an incomplete preoperative evaluation of a complex adnexal mass, followed by an inadequate surgery. It is to avoid this unwanted sequence that the IFS represents a potentially powerful tool for the gynecologic oncology surgeons in the right setting.

The IFS analysis of ovarian masses allows gynecological oncologists to perform the optimal surgery to a given patient, therefore preventing the unnecessary morbidity of excessive surgical staging in benign cases and the need for restaging procedures in early-stage malignant tumors.^12^ The IFS analysis is only valuable if it may alter the procedure that the surgeon performs at the time of the operation, which is the case of ovarian cancer surgery.

The use of IFS offers a very good diagnostic accuracy in distinguishing women with malignant and benign ovarian tumors. In contrast, for borderline ovarian tumors, IFS results in more diagnostic discrepancies.

In a study of 274 patients, the sensitivity and specificity of IFS for benign, borderline and malignant tumors were 97% and 81%, 62% and 96% and 88% and 99%, respectively. The histologic type (mucinous), tumor size (< 10 cm), the borderline component (< 10%) and the pathologist experience predicted the misdiagnosis of borderline tumors.^13^ In another study, the IFS diagnosis agreed with the PS diagnosis in 94% of all cases (98.5% for malignant tumors, 94% for benign tumors, and 78.6% for borderline tumors). The sensitivity and specificity values for malignant tumors were 93 and 99%; for borderline tumors, 61 and 99%; and for benign tumors, 98 and 93%, respectively.^14^

In a majority of studies, the sensitivity, specificity and predictive values of IFS diagnoses for benign and malignant tumors were found to be relatively high.^5^ ^6^ ^7^ ^15^ ^16^ On the other hand, the sensitivity of IFS for borderline ovarian tumors was ∼ 60% in previous reports.^14^ ^17^

In our experience, the sensitivity and specificity for benign, borderline, and malignant tumors were 100%, 66.7%, 96.9%, and 97.1%, 99.4%, 100%, respectively.

Out of the 184 IFSs reported in the present study, 98.9% of the women were submitted to the correct operative procedure at the initial surgical operation, only 0.54% of the women were under-staged at the first surgery, and 0.54% of the women were over-staged based on the IFS report.

The present study shows that IFS can contribute significantly to determine the malignant or benign nature of epithelial ovarian tumors, whereas for borderline tumors, its accuracy appears to be more dependent on the experience of the pathologist and on the characteristics of the tumor.

The main limitation of the IFS is the difficulty to obtain an accurate diagnosis of borderline ovarian tumors, mainly of the mucinous type.

Various reasons have been proposed for the relative inaccuracy of IFS in the diagnosis of borderline tumors. In a large borderline tumor, there may be only occasional foci of atypia amounting to the borderline category. On the other hand, severe atypia and/or invasion may be focal, but amounting to frank malignancy in the final reporting. Ovarian mucinous borderline tumors may contain benign, borderline and malignant areas in the same tumor. Therefore, the final reporting may require a large number of sections to be processed, an option not usually available during IFS, as it is very labor-intensive and time-consuming. It has also been suggested that it may be more difficult to diagnose borderline mucinous tumors compared with borderline serous tumors because of their larger average size.^18^

In a univariate analysis, underdiagnosis was shown to be more likely in non-serous epithelial tumors.^15^ Another study showed a 9% inaccuracy rate in serous tumors compared with 36.6% in mucinous tumors.^19^ Most false negatives occur in mucinous neoplasms and borderline tumors of various types.^12^

In summary, IFS can be of clinical use and the surgeons may feel confident enough to base appropriate surgical action upon a given result in experienced centers. Proximity between surgeons and pathologists seems advisable.

### Intraoperative Frozen Section in Uterine Tumors

Endometrial cancer is the most common malignant tumor of the genital tract worldwide.^7^

In endometrial carcinoma, the incidence of pelvic/para-aortic lymph node metastasis is related to the grade of the tumor, the depth of myometrial invasion and the presence of cervical involvement. These factors determine the type of initial surgery and the extent of the surgical staging.^20^ The IFS analysis has been used for this purpose to identify patients requiring pelvic/para-aortic lymphadenectomy.

The key finding from the study was that, in experienced hands, the IFS analysis is accurate in identifying the subgroup of patients with high-risk EC who will benefit from full surgical staging at the time of their primary surgery.

In the present study, IFS for both histologic grade and depth of myometrial invasion correlated strongly with the final pathology analysis, supporting the use of IFS as a means to guide intraoperative decisions regarding lymphadenectomy.

The depth of myometrial invasion was accurately diagnosed in 94.8% (73/77) of the cases. The inaccuracy resulted in sub-optimal intraoperative surgical care in only 3.9% (3/77) of the patients, with 2.6% and 1.3% being under and overtreated, respectively, at the time of the primary surgery.

The correlation between IFS and paraffin histology in patients with endometrial carcinoma was 96.25% (77/80). However, the three cases not detected in IFS were not surgically undertreated in the end.

Lymphadenectomy was performed in 32.5% (26/80) of the patients with malignant disease. In 5 cases (6.25%), lymphadenectomy was not performed due to technical difficulties, while 61.25% (49/80) were considered low-risk and were spared of being submitted to lymphadenectomy. Two patients (2.5%) were incorrectly diagnosed as low-risk and were not submitted to lymphadenectomy.

There is controversy about the use of IFS in the evaluation of endometrial tumors. In some studies, IFS accurately identified 90% of the patients requiring pelvic/para-aortic lymphadenectomy. Histologic grade on IFS correlated with the final diagnosis in 91.4 to 94% of the cases.^6^ ^16^ The depth of myometrial invasion was accurately reported in 80 to 95% of the cases.^2^ ^5^ ^7^ ^16^ However, other studies showed that IFS for histological grade and depth of myometrial invasion in EC correlates poorly (58%) with the final pathology analysis.^7^ In some studies, the evaluation of the depth of myometrial invasion with IFS has a sensitivity and a specificity of 74 and 95%, respectively, and this is not significantly higher than the radiological assessment.^21^ ^22^

It is important to say that, in IFS, we cannot expect a high accuracy concerning the histopathological classification of the tumor and its grade because both depend on:

1. Sampling criteria (for example, is it necessary to count the percentage of solid areas in microscopy); 2. evaluation with complementary techniques (for example, diverse histological types, mixed tumors, heterologous elements, etc.); 3. we can rarely make a diagnosis of a neuroendocrine tumor in the IFS; it may be suspected in extreme cases of very good differentiation or extremely small differentiation, but only with paraffin processing and immunophenotype study can we make the final diagnosis.

Criteria 1 and 2 are not subject to definitive observation in IFS; they imply total tumor inclusion. Neither is it allowed to freeze tissue more than necessary, because freezing alters the processing and complementary immune techniques, such as irreversibly preventing a correct histopathological diagnosis.

There are several reasons for inaccuracies related to all IFS samplings, including inadequate sampling and potential artifacts. If the tumor is macroscopically confined to the endometrium, it is difficult to reveal myometrial invasion in IFS, even with multiple cuts, leading to a sampling error.

For the group of patients with a preoperative diagnosis of CAH, endometrial carcinoma was diagnosed at the IFS study in 35.5% (11/31) of the cases, and all of them were confirmed in the final PS. However, the IFS failed to identify 2 cases of endometrial carcinoma. According to several studies, a considerable number of patients with EC can be missed on IFS. In one study, the diagnosis of EC was missed in 7/20 patients with perioperative CAH.^23^ In another study, the diagnosis was missed in 14/125 patients.^24^ It is important to reinforce that in the present study, as well as in other reports, these cases were early stage ECs without myometrial invasion, which can provide an explanation for the discrepancies in these cases, findings which, again, were not relevant to the surgical management.

## Conclusion

The IFS diagnosis has important implications regarding the type and extent of the surgery performed at the initial surgical approach. The IFS analysis plays a critical role in providing an appropriate surgical treatment and in avoiding under or overtreatment. We have found a high sensitivity and specificity in IFS in the diagnosis of ovarian tumors and in the determination of their malignant potential. Therefore, IFS should always be used when the preoperative diagnosis is not conclusive, in order to determine the extent of surgical resection. However, underdiagnosis can occur in tumors of the borderline category, especially those with a mucinous histology, which can be minimized by increased sampling on the frozen section. Our results demonstrate the reliability of IFS in identifying patients who should undergo lymphadenectomy. At our institution, high rates of agreement between IFS and PS were found for histological subtype and myometrial invasion. These are histopathological risk factors that are routinely used to guide the management of clinical, early-stage EC. Uterine type-II carcinomas and ovarian borderline tumors are the most challenging diagnoses and accounted for the majority of IFS misdiagnoses. The limitations of the present study are inherent to its retrospective nature, as well as to the relatively limited sample. Regular audits, including specific analysis of cases in which IFS and PS are different, should be conducted by both surgeons and pathologists as part of a quality assurance process for the intraoperative management of patients with suspected or proven ovarian or uterine cancer.
